# Baveno Criteria Safely Identify Patients With Compensated Advanced Chronic Liver Disease Who Can Avoid Variceal Screening Endoscopy: A Diagnostic Test Accuracy Meta-Analysis

**DOI:** 10.3389/fphys.2019.01028

**Published:** 2019-08-13

**Authors:** Zsolt Szakács, Bálint Erőss, Alexandra Soós, Péter Mátrai, Imre Szabó, Erika Pétervári, Judit Bajor, Nelli Farkas, Péter Hegyi, Anita Illés, Margit Solymár, Márta Balaskó, Patrícia Sarlós, Ákos Szűcs, József Czimmer, Áron Vincze, Gabriella Pár

**Affiliations:** ^1^Institute for Translational Medicine, Medical School, University of Pécs, Pécs, Hungary; ^2^János Szentágothai Research Center, University of Pécs, Pécs, Hungary; ^3^Institute of Bioanalysis, Medical School, University of Pécs, Pécs, Hungary; ^4^Division of Gastroenterology, First Department of Medicine, Medical School, University of Pécs, Pécs, Hungary; ^5^Hungarian Academy of Sciences-University of Szeged, Momentum Gastroenterology Multidisciplinary Research Group, Budapest, Hungary; ^6^First Department of Surgery, Semmelweis University, Budapest, Hungary

**Keywords:** fibroscan, platelets, variceal bleeding prediction, high-risk varices, diagnostic accuracy

## Abstract

**Background:** The Baveno VI Consensus Workshop defined criteria (liver stiffness measured by transient elastography <20 kPa and platelet count >150 × 10^9^ cells/L) to identify those patients with compensated advanced chronic liver diseases (cACLD) who are unlikely to have varices needing treatment (VNTs) and can safely avoid variceal screening endoscopy. This meta-analysis aimed to quantify the safety and efficacy of these criteria in suspected cACLD with liver stiffness >10 kPa and in compensated chronic liver diseases (cCLD) irrespective of liver stiffness.

**Methods:** A systematic search was conducted in nine databases for studies discussed cACLD or cCLD and tested Baveno criteria against variceal screening endoscopy. The main safety and efficacy endpoints were missed VNT rate and spared endoscopy rate (SER), respectively; calculated with the random effect model. Pooled sensitivity, specificity, and area under the curve (AUC) were calculated with the hierarchical summary receiver operating characteristic model. For all outcome measures, 95% confidence intervals were computed. Heterogeneity was tested with *I*^2^-statistics.

**Results:** The search yielded 13 studies including 4,464 patients which reported on suspected cACLD. Pooled missed VNT rate was 0.3% (0.1–0.6%; *I*^2^ = 45.5%), pooled SER was 32.8% (24.8–41.4%; *I*^2^ = 97.0%). Sensitivity, specificity, and AUC of Baveno criteria were 97% (95–98%), 41% (27–57%), and 96% (94–97%), respectively. In the subgroups of cACLD from hepatitis C and B viruses, non-alcoholic fatty liver disease/steatohepatitis, or alcohol, missed VNT rates were 0.0% (0.0–0.3%), 1.2% (0.4–2.2%), 0.0% (0.0–1.3%), or 0.0% (0.0–0.4%), while SERs were 24.2% (20.5–28.1%), 24.9% (21.7–28.4%), 38.6% (10.9–70.8%), or 27.0% (16.9–38.4%), respectively. If we expanded the study population to cCLD, 27 studies included 7,534 patients. Missed VNT rate was 0.2% (0.1–0.5%; *I*^2^ = 39.8%) with a SER of 30.5% (25.2–36.2%; *I*^2^ = 96.1%) while Se, Sp, and AUC were 97% (93–99%), 35% (27–44%), and 80% (77–84%), respectively.

**Conclusions:** The application of Baveno criteria significantly reduces the number of unnecessary variceal screening endoscopies while being safe: cACLD patients with liver stiffness <20 kPa and platelet count > 150 × 10^9^ cells/L carry a very low chance (i.e., 0.3%) of having VNTs. The criteria preserve low missed VNT rate with lower diagnostic performance among cCLD patients.

## Introduction

Liver cirrhosis is a significant cause of global health burden and responsible for more than one million deaths per year worldwide (Mokdad et al., 2014; Scaglione et al., 2015). One of the leading causes of liver-related fatalities is variceal bleeding (Garbuzenko, 2016). Esophageal varices are present in 30–40% of patients with compensated cirrhosis, while their prevalence reaches even 85% in patients with decompensated cirrhosis (Zipprich et al., 2012).

### Rationale

Prognosis of compensated chronic liver diseases (cCLD) with esophageal varices is far worse compared to that without them (Zipprich et al., 2012). The risk of variceal bleeding increases with advanced liver disease, with decompensation of the liver disease, with larger size of varices, and with the presence of red signs (North Italian Endoscopic Club for the Study and Treatment of Esophageal Varices, 1988). Early detection of varices and their treatment successfully reduce the risk of bleeding and increases life expectancy (De Franchis, 2015).

The gold standard of detection and grading of esophageal varices has remained esophagogastroduodenoscopy (EGD) (De Franchis, 2015). However, EGDs negative for varices needing treatment (VNTs) account for more than two-thirds of the cases, which number imposes a potentially avoidable economic burden on the healthcare system (Maurice et al., 2016; Augustin et al., 2017; Colecchia et al., 2018). Not to mention the risks and inconvenience of the procedure, leading to anxiety and potentially resulting in poor adherence (Ersoz et al., 2010; Simon et al., 2017). In the light of these drawbacks, the need for a non-invasive substitute for EGD is evident.

The Baveno VI Consensus Workshop (2015) defined an entity, compensated advanced chronic liver disease (cACLD), which carries the risk of clinically significant portal hypertension (De Franchis, 2015). To reduce the number of endoscopies, the Baveno VI recommendation allows that cACLD patients with a liver stiffness (LS) measured by transient elastography <20 kPa and a platelet count > 150 × 10^9^ cells/L (Baveno criteria) can avoid screening EGD. If both criteria are met, patients are at low risk (<5%) of having VNTs (De Franchis, 2015). In general, Baveno criteria aimed to rule out (and not to rule in) VNTs.

One year later, a meta-analysis summarized how LS and platelet count predict the risk of esophageal varices and varices at risk of bleeding (Marot et al., 2017). However, this meta-analysis was limited due to the available evidence because mostly conference abstracts were included and the low number of studies did not allow the restriction of study population to cACLD, nor to different etiologies. Since then, a large body of evidence has accumulated based on several high-quality diagnostic test accuracy studies (Augustin et al., 2017; Jangouk et al., 2017; Llop et al., 2017; Silva et al., 2017; Sousa et al., 2017; Bae et al., 2018; Bellan et al., 2018; Colecchia et al., 2018; Lee et al., 2018; Petta et al., 2018; Stanislas et al., 2018; Thabut et al., 2019; Tosetti et al., 2019a).

### Objectives and Research Question

The aim of this meta-analysis was to provide an up-to-date summary on the safety and efficacy of Baveno criteria (LS < 20 kPa and platelet count > 150 × 10^9^ cells/L), focusing on cACLD and cCLD of various causes.

## Methods

### Study Design

We conducted and reported this work in accordance with the Preferred Reporting Items for a Systematic Review and Meta-analysis of Diagnostic Test Accuracy Studies (2018) (McInnes et al., 2018).

### Participants, Interventions, Comparators

The population of interest included adult cases of cCLD. The diagnostic accuracy for predicting VNTs of Baveno criteria was compared to that of the standard EGD.

### Systematic Review Protocol

The protocol of this meta-analysis was registered in advance in the PROSPERO database under the registration number of CRD42018096146.

### Search Strategy

We performed a systematic search in nine medical databases (MEDLINE via PubMed, EMBASE via Ovid, CENTRAL, Web of Science, Scopus, WHO Global Health Library, ScieELO, LILACS, and ClinicalTrials.gov) from 1st January 2003, when transient elastography (Fibroscan®) became available in Europe, up to the end of May 2019 with the free-text terms *liver AND (stiffness OR elastography OR sonoelastography OR fibroscan OR platelet OR thrombocyte OR BAVENO) AND (varices OR varix OR “portal hypertension”)*. These were combined with Medical Subject Headings including *liver, hypertension portal; blood platelets, elasticity imaging techniques, varicose veins*. Apart from the time restriction, we did not apply any filters to the search.

As additional data sources, we went through the reference lists of relevant included and excluded records with hand search and sought for citing articles with Google Scholar.

### Study Selection and Data Extraction

Eligible diagnostic accuracy studies discussed adult cases of cCLD and tested the diagnostic accuracy of Baveno criteria (i.e., the index test) against EGD (i.e., the reference standard) in the detection of VNTs. The primary safety and efficacy outcomes were missed VNT rate and spared endoscopy rate (SER), respectively. Secondary outcomes included diagnostic accuracy metrics, such as sensitivity, specificity, and area under the curve (AUC) of Baveno criteria for detecting VNTs.

cCLD was defined as a chronic liver disease of Child-Pugh A or B without obvious signs of parenchymal or portal decompensation. Within cCLD, we investigated two subgroups of suspected cALCD: one contained studies with LS > 10 kPa in accordance with Baveno VI recommendations, the other contained studies using an LS cut-off between 10 and 15 kPa. VNTs were defined as per the criteria within the individual studies (medium/large esophageal varices or the presence of red signs on varices with any grade).

Records yielded by the search were combined in a reference manager software (EndNote X7.4, Clarivate Analytics, Philadelphia, PA, US). Our selection and data collection process were designed according to the Cochrane Handbook for Systematic Reviews (Higgins and Green, 2011). After removing overlaps between databases and duplicate references, the remaining records were subjected a standard three-step selection (by title, abstract, and full-text) by two review authors in duplicate, with third-party arbitration resolving discrepancies after each step. Eligible original studies (including conference abstracts, journal articles, letters, comments, and replies) were subjected to data collection onto pre-defined Excel sheets by the same review authors included in selection. Discrepancies were resolved by consensus. Technical details of data collection are described in Supplementary Appendix 1.

### Risk of Bias and Applicability Assessment

Two review authors assessed risk of bias in and applicability of the individual studies with the Quality Assessment of Diagnostic Accuracy Studies-2 (QUADAS-2) (Whiting et al., 2011). The topic-tailored items are presented in Supplementary Appendix 2.

### Quality of Evidence

The Grading of Recommendations Assessment, Development and Evaluation (GRADE) was used for estimating the quality of evidence of safety and efficacy endpoints (Guyatt et al., 2008). Since upgrading items are not validated to diagnostic test accuracy meta-analyses, we decided to assess grade of evidence along five downgrading items, that is, risk of bias, indirectness, inconsistency, imprecision, and publication bias.

### Data Analysis

We constructed 2 × 2 diagnostic tables from the data extracted. Tables consist of true positive, true negative, false positive, and false negative cells containing the number of corresponding patients. Reference and index tests were chosen to be EGD and Baveno criteria (cut-off for every study: a platelet count > 150 × 10^9^ cells/L and an LS <20 kPa), respectively. Missed VNT rate [false negative cases/total number of patients] and SER [(true negative + false negative cases)/total number of patients] were calculated by using the random effects model with the Der-Simonian Lard estimation (Cales et al., 2018). Sensitivity (Se), specificity (Sp), and AUC were calculated by using the hierarchical summary receiver operating characteristic (HSROC) model. 95% confidence intervals (CIs) and predictive intervals (PIs) were used in all analyses.

Heterogeneity was examined with *I*^2^- and *chi*^2^-test and explored with subgroup analysis and metaregression. Pre-planned subgroup analyses were carried out for patients with suspected cACLD (with an LS cut-off of 10 kPa and between 10 and 15 kPa), etiology (hepatitis B and C viruses [HBV and HCV, respectively], non-alcoholic fatty liver disease/non-alcoholic steatohepatitis [NAFLD/NASH], and alcoholic liver disease [ALD]), publication type (journal article vs. conference abstract), and prospectivity of the study. Meta-regression was performed to analyze the effects of 14 variables on missed VNT rate and SER.

Egger's test was performed and funnel plots were constructed to test small-study. If *p* ≥ 0.1, publication bias is unlikely to occur in the sample.

Further details are reported in Supplementary Appendix 1.

## Results

### Study Selection and Characteristics

**Figure 1:**
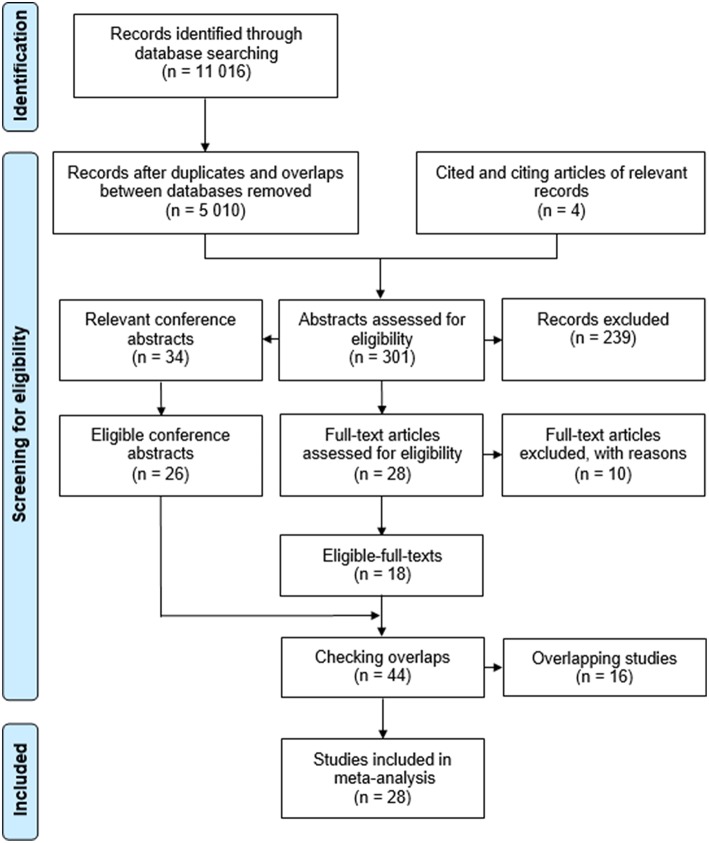
Flowchart of the meta-analysis.

Figure 1 displays the flow of work. The search yielded an overall 11,016 records in databases (MEDLINE: 1,410, EMBASE: 4,738, CENTRAL: 212, Web of Science: 1,713, Scopus: 2,708, WHO Global Health Library: 166, ScieELO: 24, LILACS: 26, and ClinicalTrials.gov: 19). After careful selection, 44 potentially eligible studies were subjected for inclusion, 16 of which (15 conference abstracts and one journal article) were excluded due to overlapping study populations (Supplementary Appendix 3). For the same reason, only subgroups of two multicenter studies (Augustin et al., 2017; Petta et al., 2018) were eligible for inclusion (Supplementary Appendix 3). Finally, 17 journal articles and 11 conference abstracts were included (Perazzo et al., 2015; Chang et al., 2016; Maurice et al., 2016; Arriero et al., 2017; Augustin et al., 2017; Jangouk et al., 2017; Llop et al., 2017; Perez Ferrer et al., 2017; Silva et al., 2017; Sousa et al., 2017; Bae et al., 2018; Bai et al., 2018; Bellan et al., 2018; Cales et al., 2018; Colecchia et al., 2018; Kew et al., 2018; Lee et al., 2018; Levick et al., 2018; Matsui et al., 2018; McDowell et al., 2018; Ng et al., 2018; Pawar et al., 2018; Petta et al., 2018; Stanislas et al., 2018; Tadkalkar et al., 2018; Moctezuma-Velazquez et al., 2019; Thabut et al., 2019; Tosetti et al., 2019a,b). All the 28 studies provided data on missed VNT rate and SER but one conference abstract (Tosetti et al., 2019b) was used only in subgroup analysis by etiology due to overlapping study population with a full-text article in the main analysis (Tosetti et al., 2019a). A total of 26 studies were eligible for HSROC analysis. Table 1 summarizes the characteristics of the studies included. The most common underlying pathologies were HCV and alcohol. Prevalence of VNTs ranged from 4 to 33% in study populations suggesting a wide range of disease severity.

**Table 1:** Characteristics of the studies included.

**References**	**Setting**	**Population**	**Etiology**	**No. of patients**	**Prevalence of VNTs**	**TP**	**FP**	**FN**	**TN**
Arriero et al., 2017^*^	Spain, single-center (retrospective; 2013 January−2016 April)	cACLD (LS > 12 kPa)	Viral: 52.4%, other: 47.6%	234	5%	12	182	0	40
Augustin et al., 2017	Spain, France, Romania, and Canada, multi-center (retrospective; 2014–2015)	cACLD (LS ≥ 10 kPa)	HCV	117	7%	9	79	0	29
Bae et al., 2018	Korea, single-center (retrospective; 2012 July−2016 December)	cACLD (LS ≥ 10 kPa)	HBV: 58.9%, HCV: 16.3%, ALD: 18.8%, NAFLD: 6%	282	20%	52	152	3	75
Bai et al., 2018^*^	New Zealand, single-center (not reported)	cACLD (LS ≥ 10 kPa)	HCV: 40%, NAFLD: 24.9%, HBV: 15.6%	205	10%	20	131	0	54
Bellan et al., 2018	Italy (not reported)	cCLD (LS ≥ 6 kPa)	HCV	147	11%	16	97	1	33
Chang et al., 2016^*^	Singapore, single-center (retrospective; 2006–2012)	cACLD (LS > 12 kPa)	Viral: 55%, other: 45%	173	8%	11	128	3	31
Colecchia et al., 2018	Italy and Switzerland, three-center (retrospective; 2012–2016 and prospective; 2017 September−2018 February)	cACLD (LS ≥ 10 kPa)	HCV: 76.6%, HBV: 5.8%, ALD: 8.8%, NAFLD/NASH: 3.1%, autoimmune: 0.5%, miscellaneous: 5.2%	613	19%	114	378	1	120
Jangouk et al., 2017	USA and Italy, double-center (retrospective; 2010 May−2016 April and 2014 February−2016 April)	cACLD (LS ≥10 kPa)	HCV: 70.6%, ALD: 12.5%, NAFLD: 7.7%, other: 9.2%	262	12%	31	174	0	57
Kew et al., 2018^*^	Singapore (retrospective; 2013–2015)	cACLD (LS ≥ 10 kPa)	HBV: 30%, NASH: 24.4%, HCV: 17.7%,	164	18%	28	86	2	48
Lee et al., 2018	Korea; 4-center (retrospective; 2014 January−2017 December)	cACLD (LS ≥ 10 kPa)	HBV: 39.7%, HCV: 12.1%, ALD: 29.2%, other: 19.0%	1218	20%	243	662	6	307
Levick et al., 2018^*^	UK (not reported)	cACLD	ALD: 35%, NAFLD: 22%, viral: 16%, other: 27%	45	20%	9	29	0	7
Llop et al., 2017	Spain, single-center (retrospective; 2013 September−2015 September)	cACLD (LS ≥ 10 kPa)	HCV: 85.1%, HCV and ALD: 3.1%, HBV: 3.7%, autoimmune: 3.1%, NASH: 3.7%, other: 1.2%	161	Not reported	Not reported	Not reported	0	54
Maurice et al., 2016	UK, double-center (retrospective; 2006 November−2015 September)	cACLD (LS ≥ 10 kPa)	HCV: 55%, HBV/HDV: 8%, ALD: 13%, NAFLD: 14%, other: 11%	310	5%	13	195	2	100
Matsui et al., 2018	Japan; double-center (retrospective; 2013 April−2016 December)	cCLD	NAFLD: 39%, HCV: 39%, ALD: 9%, HBV: 6%; PBC: 6%	384	3%	46	104	2	223
McDowell et al., 2018^*^	UK; single-center (retrospective)	cCLD	Not reported	82	Not reported	Not reported	Not reported	0	24
Moctezuma-Velazquez et al., 2019	UK, Spain, Canada, Switzerland; 4-center (retrospective; 2009 March−2017 December)	cACLD (LS > 10 kPa)	PBC: 65%, PSC: 35%	227	13%	30	115	0	82
Ng et al., 2018^*^	Australia; single-center (retrospective; 2011 May−2016 June)	cACLD (LS > 12 kPa)	NAFLD: 60%, ALD: 40%	85	8%	7	53	0	25
Pawar et al., 2018^*^	India (prospective)	cACLD (LS > 10 kPa)	NASH: 47.72%, HBV: 20.45%, ALD: 13.63%, others: 11,36%, HCV: 5.68%, HBV/HCV: 1.13%	88	31%	24	6	3	55
Perazzo et al., 2015	Brazil (prospective)	cACLD	Not reported	97	14%	14	62	0	21
Perez Ferrer et al., 2017^*^	Spain, single-center (not reported)	cACLD (LS > 15 kPa)	HCV	32	16%	1	14	4	13
Petta et al., 2018	Italy, France, Canada, UK, Hong Kong, and Switzerland, ten-center (retrospective)	cACLD (LS > 11 kPa or >11.5 kPa)	NAFLD	639	11%	67	338	6	228
Silva et al., 2017	Portugal, single-center (retrospective; 2009 September−2015 October)	cACLD (LS > 12 kPa)	HCV: 78.4%, ALD: 8.2%, HBV: 3.1%, biliary: 3.1%, other: 7.2%	97	14%	14	72	0	11
Sousa et al., 2017	Portugal, single-center (retrospective; 2013 January−2015 December)	cCLD	HCV: 66%, HCV/HIV: 16%, ALD: 12%, HBV: 4%, other: 5%	104	9%	9	47	0	48
Stanislas et al., 2018	Côte d'Ivoire, double-center (prospective; 2016 January−2016 July)	cACLD (LS ≥ 11 kPa)	HBV	60	33%	20	28	0	12
Tadkalkar et al., 2018^*^	India (2016 September−2017 August)	cACLD (LS > 10 kPa)	HBV, HCV, NASH, ALD	375	22%	80	29	4	262
Thabut et al., 2019	France; 35-center (retrospective; until 2015 December)	cCLD	HCV: 81%, HBV: 16.6%, HCV/HBV: 2.4%	891	8%	69	601	3	298
Tosetti et al., 2019a	Italy; single-center (retrospective; 2010 February−2016 January)	cACLD (LS ≥ 10 kPa)	HCV: 69%, HBV: 10%, NASH: 15%, ALD: 6%	442	7%	31	325	0	86
Tosetti et al., 2019b^*^	Italy (retrospective)	cACLD (LS ≥ 10 kPa)	HCV	192	4%	8	141	0	43

* *Conference abstracts. ALD, alcoholic liver disease; cACLD, compensated advanced chronic liver disease; cCLD, compensated chronic liver disease; FN, false negative; FP, false positive; HBV, hepatitis B virus; HCV, hepatitis C virus; HDV, hepatitis D virus; HIV, human immunodeficiency virus; LS, liver stiffness; NAFLD, non-alcoholic fatty liver disease; NASH, non-alcoholic steatohepatitis; PBC, primary biliary cirrhosis; PSC, primary sclerotizing cholangitis; TN, true negative; TP, true positive; US, ultrasound; VNT, varices needing treatment*.

### Risk of Bias and Applicability

**Figure 2:**
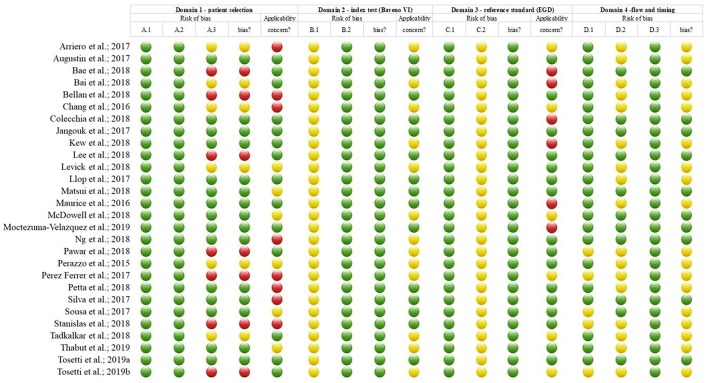
Risk of bias and applicability assessment with QUADAS-2. Domains A-D cover patient selection, index test (i.e., the Baveno criteria), reference standard (i.e., esophagoscopy), and flow and timing, respectively. All domains contain sets of questions concerning risk of bias (columns A1–A3, B1–B2, C1–C2, and D1–D3 in the figure), answered with yes, no, or uncertain denoted by a green tick, a red x, or a blue question mark, respectively; summarized as an overall risk of bias for each domain (columns RoB in the figure; green tick, red x, and blue question mark represent low, high, and uncertain risk of bias). Domains A-C include an applicability item each; green tick, red x, and blue question mark represent low, high, and uncertain concerns regarding applicability, respectively. For further details of the assessment, see Supplementary Appendix 2.

Risk of bias and applicability of the studies included is summarized in Figure 2.

### Performance of Baveno Criteria Among cACLD Patients With an LS Cut-Off of 10 kPa

**Figure 3:**
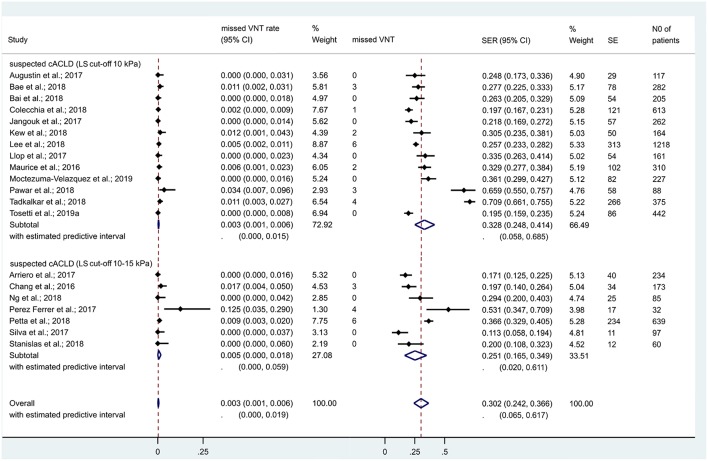
Missed VNT rate and spared endoscopy rate with the Baveno criteria among patients with compensated advanced chronic liver disease. cACLD, compensated advanced chronic liver disease; CI, confidence interval; SE, spared endoscopies; SER, spared endoscopy rate; VNT, varices needing treatment.

**Figure 4:**
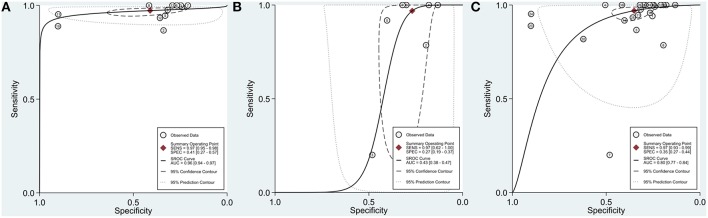
Summary receiver operating characteristic (SROC) curves. **(A)** Compensated advanced chronic liver disease (with an LS cut-off of 10 kPa). **(B)** Compensated advanced chronic liver disease (with an LS cut-off between 10 and 15 kPa). **(C)** Compensated chronic liver disease. AUC, area under the curve; LS, liver stiffness; SENS, sensitivity; SPEC, specificity.

In the 13 studies which discussed suspected cACLD (with an LS cut-off of 10 kPa), 4,464 patients were included. Missed VNT rate was 0.3% [CI 0.1–0.6% (*I*^2^ = 45.5%, *p* = 0.037)] with a SER of 32.8% [CI 24.8–41.4% (*I*^2^ = 97%, *p* < 0.001)] (Figure 3). Se, Sp, and AUC of Baveno criteria were 97% [CI 95–98%], 41% [CI 27–57%], and 96% [CI 94–97%], respectively (Figure 4A).

**Figure 5:**
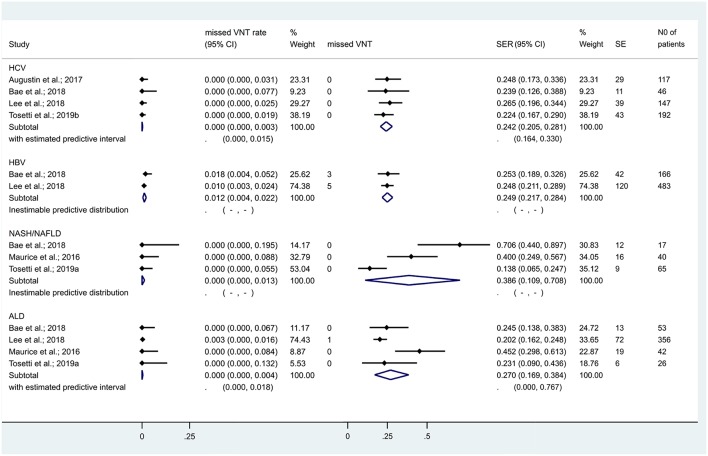
Missed VNT rate and spared endoscopy rate with the Baveno criteria among patients with compensated advanced chronic liver disease of various etiologies. We failed to investigate heterogeneity and calculate predictive intervals for the subgroups of HBV and NASH/NAFLD due to the low number of studies included. ALD, alcoholic liver disease; cACLD, compensated advanced chronic liver disease; CI, confidence interval; HCV, hepatitis C virus; HBV, hepatitis B virus; NASH/NAFLD, non-alcoholic steatohepatitis/non-alcoholic fatty liver disease; SE, spared endoscopies; SER, spared endoscopy rate; VNT, varices needing treatment.

When we restricted the analysis to cACLD with HCV, HBV, NAFLD/NASH, and ALD, missed VNT rates were 0.0% [CI 0.0–0.3%], 1.2% [CI 0.4–2.2%], 0.0% [CI 0.0–1.3%], and 0.0% [CI 0.0–0.4%], while SERs were 24.2% [CI 20.5–28.1%], 24.9% [CI 21.7–28.4%], 38.6% [CI 10.9–70.8%], and 27.0% [CI 16.9–38.4%], respectively (Figure 5). The number of studies did not allow us to calculate other diagnostic metrics reliably.

Normalized frequencies (Se: 97% and Sp: 41%) assuming a prevalence of VNTs to be 5, 10, 15, 20, and 25% are reported in Table 2.

**Table 2:** Normalized frequencies for a sensitivity of 97% and a specificity of 43%.

**In a sample population of 1,000 subjects with cACLD (with an LS cut-off 10 kPa)**					
Prevalence of VNTs	5%	10%	15%	20%	25%
VNTs (*n*)	50	100	150	200	250
Missed VNTs (*n*)	1.5	3	4.5	6	7.5
Still unnecessary endoscopies (*n*)	560.5	531	501.5	472	442.5
Spared endoscopies (*n*)	391	372	353	334	315

*cACLD, compensated advanced chronic liver disease; LS, liver stiffness; VNTs, varices needing treatment*.

### Performance of Baveno Criteria Among cACLD Patients With an LS Cut-Off Between 10 and 15 kPa

In the 7 studies which discussed suspected cACLD (with an LS cut-off between 10 and 15 kPa), missed VNT rate was 0.5% [CI 0.0–1.8% (*I*^2^ = 65.1%, *p* = 0.009)] with a SER of 25.1% [CI 16.5–34.9% (*I*^2^ = 91.6%, *p* < 0.001)] (Figure 3). Se, Sp, and AUC of Baveno criteria were 97% [CI 62–100%], 27% [CI 19–37%], and 43% [CI 38–47%], respectively (Figure 4B). The number of studies did not allow us to perform subgroup analysis by etiology.

### Performance of Baveno Criteria Among cCLD Patients

**Figure 6:**
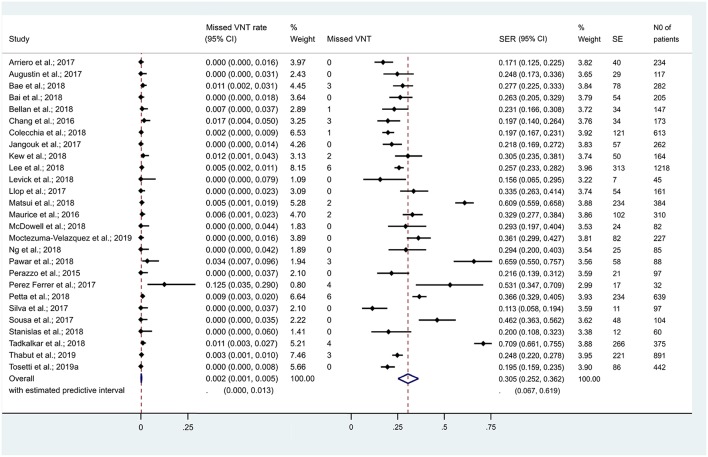
Missed VNT rate and spared endoscopy rate with the Baveno criteria among patients with compensated chronic liver disease. CI, confidence interval; SE, spared endoscopies; SER, spared endoscopy rate; VNT, varices needing treatment.

If we expanded the coverage of the meta-analyis to patients with cCLD (irrespective of LS), 27 studies reported on 7,534 cCLD patients. Missed VNT rate was 0.2% [CI 0.1–0.5% (*I*^2^ = 39.8%, *p* = 0.019)] with a SER of 30.5% [CI 25.2–36.2% (*I*^2^ = 96.1%, *p* < 0.001)] (Figure 6). Se, Sp, and AUC of Baveno criteria were 97% [CI 93–99%], 35% [CI 27–44%], and 80% [CI 77–84%], respectively (Figure 4C).

**Figure 7:**
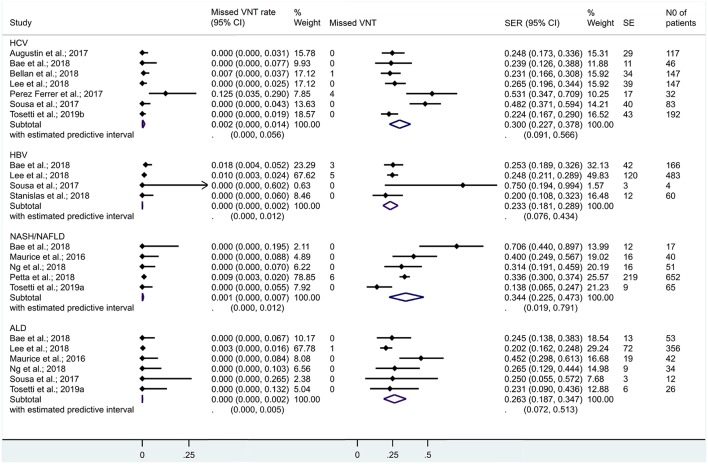
Missed VNT rate and spared endoscopy rate with the Baveno criteria among patients with compensated chronic liver disease of various etiologies. ALD, alcoholic liver disease; CI, confidence interval; HCV, hepatitis C virus; HBV, hepatitis B virus; NASH/NAFLD, non-alcoholic steatohepatitis/non-alcoholic fatty liver disease; SE, spared endoscopies; SER, spared endoscopy rate; VNT, varices needing treatment.

When we restricted the analysis to cCLD from HCV, HBV, NAFLD/NASH, and ALD, missed VNT rates were 0.2% [CI 0.0–1.4%], 0.0% [CI 0.0–0.2%], 0.1% [CI 0.0–0.7%], and 0.0% [CI 0.0–0.2%], while SERs were 30.0% [CI 22.7–37.8%], 23.3% [CI 18.1–28.9%], 34.4% [CI 22.5–47.3%], and 26.3% [CI 18.7–34.7%], respectively (Figure 7). Se, Sp, and AUC of Baveno criteria were 99% [CI 57–100%], 32% [CI 25–41%], and 47% [CI 43–51%] for cCLD from HCV and 99% [CI 82–100%], 32% [CI 25–40%], and 82% [CI 78–85%] for cCLD from ALD, respectively. The number of studies did not allow us to calculate other diagnostic metrics reliably for cCLD from NASH/NAFLD or HBV.

### Exploration of Statistical Heterogeneity

Effects of subgroups by etiology are discussed above (Figures 5, 7). When we removed conference abstracts from the analysis, the heterogeneity reduced (*I*^2^ = 0.0%, *p* = 0.564) while missed VNT rate was maintained at 0.2% [CI 0.1–0.4%]. This effect was not manifested in efficacy, where heterogeneity persisted at *I*^2^ = 94.7%, *p* < 0.001 with slightly reduced SER (28.2% [CI 23.2–33.6%]). The separate analysis of the four prospective studies did not reduce heterogeneity.

On metaregression, none of the potential modifiers of Baveno criteria had a significant effect on missed VNT rate. Interestingly, there was a significant positive correlation of SER with age, albumin level, and the proportion of NAFLD/NASH in the study population, while a significant negative correlation with the proportion of viral liver disease in the study population and with the alanine and aspartate aminotransferase levels (Table 3).

**Table 3:** Metaregression analyses with the potential modifiers of missed VNT rate and spared endoscopy rate.

**Explanatory variable**	**No. of observations**	**Coefficient (95% CI)**	***P-*value**
**Missed VNT rate**			
Age (year)	16	0.001 (−0.009 to 0.095)	0.981
Albumin (g/dL)	14	0.013 (−0.166 to 0.168)	0.987
Alcoholic etiology (% of total)	19	0.000 (−0.002 to 0.003)	0.956
ALT (U/l)	14	−0.001 (−0.012 to 0.011)	0.919
AST (U/l)	13	0.000 (−0.028 to 0.028)	0.984
Bilirubin (mg/dL)	13	−0.034 (−0.135 to 0.128)	0.956
BMI (kg/m^2^)	14	−0.010 (−0.238 to 0.219)	0.929
Creatinine (mg/dL)	12	−0.007 (−0.330 to 0.317)	0.992
Gender (male%)	20	0.000 (−0.003 to 0.003)	0.959
INR	12	−0.043 (−0.908 to 0.823)	0.915
Liver stiffness (kPa)	21	−0.001 (−0.004 to 0.040)	0.952
MELD score	10	−0.017 (−0.040 to 0.370)	0.922
NAFLD/NASH (% of total)	17	0.000 (−0.002 to 0.002)	0.889
Platelet count (10^9^ cells/L)	21	0.000 (−0.013 to 0.012)	0.961
Viral etiology (% of total)	22	0.000 (−0.001 to 0.001)	0.935
**SER**			
Age (year)	16	0.019 (0.012 to 0.037)	0.038^*^
Albumin (g/dL)	14	0.336 (0.004 to 0.667)	0.048^*^
Alcoholic etiology (% of total)	19	0.015 (−0.006 to 0.009)	0.701
ALT (U/l)	14	−0.003 (−0.005 to −0.001)	0.047^*^
AST (U/l)	13	−0.006 (−0.010 to −0.001)	0.028^*^
Bilirubin (mg/dL)	13	0.084 (−0.058 to 0.225)	0.219
BMI (kg/m^2^)	14	−0.038 (−0.083 to 0.007)	0.093
Creatinine (mg/dL)	Conditions of the analysis were not met		
Gender (male%)	20	−0.002 (−0.007 to 0.004)	0.554
INR	12	−0.753 (−1.831 to 0.325)	0.151
Liver stiffness (kPa)	21	−0.008 (−0.018 to 0.003)	0.134
MELD score	10	−0.013 (−0.059 to 0.033)	0.541
NAFLD/NASH (% of total)	17	0.005 (0.001 to 0.009)	0.001^*^
Platelet count (10^9^ cells/L)	21	0.003 (-0.001 to 0.005)	0.050
Viral etiology (% of total)	22	−0.002 (−0.004 to −0.000)	0.046^*^

* *Indicates a probability value (p) <0.05. AST, aspartate aminotransferase; ALT, alanine aminotransferase; BMI, body mass index; CI, confidence interval; INR, international normalized ratio; MELD, model for end-stage liver disease; NASH/NAFLD, non-alcoholic steatohepatitis/non-alcoholic fatty liver disease; VNT, varices needing treatment*.

### Publication Bias

Neither the Egger's tests nor the visual assessment of the symmetry of the funnel plots indicated the presence of publication bias (Supplementary Appendix 4).

## Discussion

A large number of studies focused on how safely and effectively Baveno criteria rule out VNTs in cACLD, so we performed this meta-analysis to investigate diagnostic metrics of Baveno criteria regarding VNTs. Our meta-analysis testifies that, though Baveno criteria safely rule out VNTs in cACLD (missed VNT rate: 0.3%), SER remains still relatively low (32.8%). Based on our results, substantially <5% of VNTs would have been missed by using the criteria (De Franchis, 2015). In other words, diagnostic metrics of Baveno criteria proved to be excellent in cACLD (LS <10 kPa) (Figure 4A). However, the criteria are limited: although they seem to be adequate for ruling out VNTs, a large number of EGD is still unnecessary (Table 2). GRADE approach is summarized in Table 4.

**Table 4:** Summary of findings table.

**Population:** patients suffering from cACLD (with an LS cut-off of 10 kPa)					
**Index test:** Baveno VI criteria for ruling out VNTs (LS < 20 kPa and platelet count >150 × 10^9^ cells/L)					
**Reference standard:** Variceal screening endoscopy					
**Outcomes:** Safety (i.e., missed VNT rate) and efficacy (i.e., SER)					
**Outcomes**	**No. of studies (patients)**	**Point estimate (95% confidence interval)**	**Importance**	**Quality of evidence**	**Comments**
Missed VNT rate	13 (4,464)	0.3% 0.1–0.6%	Critical	•••° moderate	Downgraded for risk of bias
SER	13 (4,464)	32.8% 24.8–41.4%	Important	••°° low	Downgraded for risk of bias and inconsistency

*cACLD, compensated advanced chronic liver disease; LS, liver stiffness; SER, spared endoscopy rate; VNTs, varices needing treatment*.

A previous meta-analysis focused on partly overlapping questions (Marot et al., 2017). Its available evidence was humble: only two out of ten reports were fully published articles, other data were collected from conference material including personal communication (“gray literature”). In contrast, our study focusing strictly on VNTs included 28 reports, of them 17 were journal articles (“white,” peer-reviewed literature) (Table 1). In addition, we had available data to investigate the safety and efficacy of Baveno criteria in cCLD and cACLD from different etiologies (Figures 5, 7). To preserve transparency, we decided not to extract gray data from the previous meta-analysis if the original record with a full dataset was not available online.

While the previous meta-analysis found a negative predictive value of 96.9% for VNTs, the increased sample size and the higher quality of studies validating Baveno criteria resulted in a missed VNT rate of 0.3% among cACLD with LS > 10 kPa and 0.2% among cCLD irrespective of LS. Results were consistent in the subgroups defined by etiology of the liver disease. The exclusion of conference abstracts homogenized the data.

Results of meta-regressions were surprising (Table 3). While the safety endpoint (i.e., missed VNT rate) remained unaffected, the efficacy endpoint (i.e., SER) showed a significant correlation with several variables. In viral liver disease, elastography may overestimate LS; thereby, leading to the negative correlation of the proportion of viral liver disease, alanine, and aspartate amino transferases with SER. The positive correlation between SER and albumin level may reflect the severity of the liver disease because, as proved by calculating normalized frequencies (Table 2), the higher the proportion of VNTs in a population, the less effective Baveno criteria are. However, we cannot explain the positive correlation between the proportion of NAFLD/NASH and SER. Indeed, the criteria work well in this subgroup as well. We must be cautious when interpreting these results, since a metaregression from population-level data is strongly limited.

Our meta-analysis has several strengths, most importantly the rigorous methodology. We performed a systematic search followed by reproducible selection and data extraction. Risk of bias assessment was performed with the use of the recent gold standard methodology, the QUADAS-2 (Whiting et al., 2011). Meta-analytical calculations were performed by experienced biostatisticians. The grade of evidence of our statements was quantified with the GRADE approach (Table 4; Guyatt et al., 2008). Small-study effect was unlikely to distort our results, as revealed by the symmetry of funnel plots with the confirmatory results of the Egger's tests (Supplementary Appendix 4).

Although there was substantial statistical heterogeneity in some analyses, we identified several potential confounding factors. When we set up subgroups by etiology of the liver disease, heterogeneity reduced to 0% for missed VNT rate in cCLD from HBV, NAFLD/NASH, and HCV and in cACLD (LS <10 kPa) from HCV and ALD. However, we have to acknowledge that if less than four studies were included, the statistical model did not allow the computation of *I*^2^ values (Figure 5). About one-third of the papers included were conference abstracts; elimination of these from the analysis reduced the heterogeneity substantially while it did not change the main associations. Metaregression demonstrated the potential modifying effects of frequently measured clinical variables as well (Table 3). However, these results should rather be considered as explanatory ones, since none of the studies included analyzed the effect of continuous variables on missed VNT rate and SER.

Risk of bias and inapplicability may distort our results as demonstrated by the QUADAS-2 (Figure 2). Since most studies were retrospective, blinding of the operators, and endoscopists was probably not introduced. The time elapsed between the EGD and transient elastography was not reported in few studies, while that between EGD and the measurement of platelet count was lacking in most studies. Ideally, LS and platelet count should be measured at the same time than variceal assessment; however, studies were permissive because 3–12 months difference between measurements were almost always tolerated. Four studies reported the use of M-probes exclusively, while only five studies reported the use of M- and XL-probes depending on nutritional status. This raises concerns about the number of unsuccessful LS measurements, imposing the risk of selection bias (Ji et al., 2014). Poor reporting of methodological details, particularly on elastography, was a common issue as well (Figure 5).

Elastography has its own drawbacks, as well. In routine clinical practice, unreliable results are obtained in nearly one in five cases, which are usually associated with obesity and are dependent on the operators' experience (Castera et al., 2010). False positivity is often associated with acute hepatitis, extra-hepatic cholestasis, liver congestion, prior food intake, and excessive alcohol intake (European Association for Study of Liver Asociacion Latinoamericana para el Estudio del Higado, 2015). Accordingly, inclusion and exclusion criteria involving these factors mildly varied across studies, restricting the applicability of our findings. The Baveno VI Consensus Workshop recommended that transient elastography should be performed in fasting condition with attention to alanine transaminase flares and should be repeated on two different days to avoid misclassification (De Franchis, 2015).

Combining the criteria with spleen stiffness measurements (with a cut-off of spleen stiffness ≤ 46 kPa) resulted in a SER of 43.8% with <5% missed VNT rate (Colecchia et al., 2018). The combination seems attractive because no further instruments (such as abdominal ultrasound) is required. Nevertheless, we must be aware of its limitation, the high rate of unsuccessful spleen stiffness measurements (15–20%) (Berzigotti, 2017).

Augustin et al. proposed that the expanded Baveno criteria (LS < 25 kPs and platelet count > 110,000/μl) may outperform the original Baveno criteria since it spares 40% of variceal screening endoscopies with a missing VNT rate of 1.6% (Augustin et al., 2017). Since, multiple studies attempted to validate these criteria with various success (Petta et al., 2018; Calvaruso et al., 2019; Dajti et al., 2019; Moctezuma-Velazquez et al., 2019; Tosetti et al., 2019a). However we must be aware that the higher SER with the application of the expanded Baveno criteria may be associated with an increased number of missed VNTs (i.e., false negative cases in the 2 × 2 diagnostic table) because the negative predictive value of the criteria is dependent on the pre-test probability of VNTs (i.e., a more severe liver disease with higher VNT rate will be associated with a lower negative predictive value of the criteria).

## Conclusions

### Implications for Practice

Low missed VNT rate of Baveno criteria suggests that only a minority of cases are misclassified who miss receiving prophylactic treatment for variceal bleeding. Missed VNTs account for 1–7.5 cases of 1,000 EGDs among cACLD patients. The lower the prevalence of VNTs, the fewer VNTs are missed; therefore, our results support the application of Baveno criteria among cACLD patients. However, the criteria remain safe with lower efficacy among cCLD patients irrespective of LS. Chance of misclassification can be minimized by annually repeated transient elastography performed by the same experienced operator and platelet count assessment that could indicate endoscopy on time (De Franchis, 2015; Perazzo et al., 2015). Our meta-analysis proves the safety of Baveno criteria in ruling out VNTs among cACLD patients while they help avoid one-third of screening variceal endoscopies. We found that Baveno criteria are applicable in HCV- and HBV-related liver disease and ALD, and also in NAFLD, but further studies are needed to validate these results.

### Implications for Research

Accuracy metrics should be considered surrogate endpoints. A randomized controlled trial with hard outcomes, such as mortality and variceal bleeding, providing definitive proof of safety, and effectivity of decision-making by Baveno criteria is warranted. We also emphasize the need for finding further non-invasive tests predicting VNT, not only to increase the number of spared endoscopy but also for patients who are lacking reliable LS and, consequently, where Baveno criteria cannot be applied.

In conclusion, our meta-analysis supports the application of Baveno criteria in cACLD to exclude VNTs, saving financial assets for health care by reducing the number of unnecessary screening endoscopies by 32.8%. Addition of serological or radiological markers to Baveno criteria may improve the accuracy of diagnosing varices. Although sparing one-third of EGDs seems cost-effective, application of Baveno criteria still suffers from a high rate of unnecessary endoscopies (442.5–560.4 cases per 1,000 EGDs). One possible option to reduce this number is changing the cut-off values (e.g., the extended Baveno criteria, risking an increasing missed VNT rate) (Bae et al., 2018) or combination of LS and platelet count with other diagnostic modalities, such as serum fibrosis markers or spleen diameter measured by ultrasound (Abraldes et al., 2016).

## Data Availability

All datasets analyzed for this study are included in the manuscript and the Supplementary Files.

## Author Contributions

ZS, GP, PH, and AV conceptualized and designed the study. EP, AI, IS, and JB acquired data and interpreted the study results. PM, AS, and NF provided biomedical statistical expertise and drafted the manuscript. MS, MB, PS, AS, and JC interpreted the results and drafted the manuscript. BE, ZS, GP, and AV critically revised the manuscript for important intellectual content. All authors read and approved the final manuscript.

### Conflict of Interest Statement

The authors declare that the research was conducted in the absence of any commercial or financial relationships that could be construed as a potential conflict of interest. The handling editor declared a past collaboration with one of the authors PH.

## Supplementary Material

Supplementary Appendix 1 Methods: data collection and statistical analysis.

Supplementary Appendix 2 Risk of bias and applicability assessment with the QUADAS-2.

Supplementary Appendix 3 Excluded studies with rationales of exclusion.

Supplementary Appendix 4 Publication bias: funnel plots and results of the Egger's tests.

## Abbreviations

ALD: alcoholic liver disease
AUC: area under the curve
cACLD: compensated advanced chronic liver disease
cCLD: compensated advanced chronic liver disease
CI: confidence interval
EGD: esophagogastroduodenoscopy
GRADE: Grading of Recommendations Assessment, Development and Evaluation
HBV: hepatitis B virus
HCV: hepatitis C virus
LS: liver stiffness
NAFLD/NASH: non-alcoholic fatty liver disease/non-alcoholic steatohepatitis
QUADAS-2: Quality Assessment of Diagnostic Accuracy Studies-2
Se: sensitivity
SER: spared endoscopy rate
Sp: specificity
VNTs: varices needing treatment.
