# Supplementary material for: Baveno Criteria Safely Identify Patients With Compensated Advanced Chronic Liver Disease Who Can Avoid Variceal Screening Endoscopy: A Diagnostic Test Accuracy Meta-Analysis
Source: Front Physiol. 2019 Aug 13;10:1028. doi: 10.3389/fphys.2019.01028 (PMC6711320; doi:10.3389/fphys.2019.01028)
Supplement: Supplementary Appendix 1 — Methods: data collection and statistical analysis. [file Table_1.docx]

**Data collection**

List of collected data were, as follows: publication data, setting (country, number of centers, recruitment period, prospective/retrospective recruitment, consecutive/random/selected population, study design), total number of patients, 2x2 diagnostic contingency tables for both EVs and VNTs, inclusion and exclusion criteria, etiology of liver diseases, definition of varices needing treatment, age, gender, liver stiffness, platelet count, body mass index, aspartate aminotransferase, alanine aminotransferase, bilirubin, albumin, international normalized ratio, creatinine, Model For End-Stage Liver Disease Score, Child score, timing of measurements (liver stiffness, platelet count, and screening endoscopy), technical details on elastography, attrition and missing data, and blinding.

Finishing data collection, a third review author thoroughly revised the collected data and the original publications (paying close attention to sites and the time period of recruitment) to link the overlapping cohorts of patients together. Definitive or highly suspect overlaps between study populations resulted in the exclusion of the whole study or subgroups of patients from analysis to prevent overrepresentation. In these cases, studies with the highest number of participants were included in analysis. If equal size of study population occurred, full-text articles were preferred to conference abstracts. We did not contact the authors of original articles for further information during selection and data collection.

**Statistical analysis**

Analysis was carried out with Stata 14 SE (StataCorp, the US).

An I^2^ of 0%-40%, 30%-60%, 50%-90%, and 75%-100% represented not important, moderate, substantial, and considerable between-study heterogeneity with *p*<0.10 indicating statistical significance.

Explanatory variables in meta regression included liver stiffness (kPa), platelet count (G/l), age (year), body mass index (kg/m2), aspartate aminotransferase (U/l), alanine aminotransferase (U/l), bilirubin (mg/dL), albumin (g/dl), international normalized ratio, creatinine (mg/dL), and Model for End-Stage Liver Disease Score. Analysis was performed if at least ten pieces of data were available for analysis. Data reporting on at least 90% of the population of that reported in 2x2 tables were considered to be eligible for meta-regressions (i.e., 10% attrition or missing data were tolerated). We report the correlation coefficient with the corresponding p-value (results with a p<0.05 were considered statistically significant).

Normalized frequencies of false negative and false positive cases (regarding VNT) were calculated with the pre-test probabilities of 5%, 10%, 15%, 20%, and 25%. For these calculations, pooled sensitivity and specificity were used.
