# Supplementary material for: Baveno Criteria Safely Identify Patients With Compensated Advanced Chronic Liver Disease Who Can Avoid Variceal Screening Endoscopy: A Diagnostic Test Accuracy Meta-Analysis
Source: Front Physiol. 2019 Aug 13;10:1028. doi: 10.3389/fphys.2019.01028 (PMC6711320; doi:10.3389/fphys.2019.01028)
Supplement: Supplementary Appendix 2 — Risk of bias and applicability assessment with the QUADAS-2. [file Table_2.docx]

**Risk of bias and applicability assessment**

A tool dedicated to assessing diagnostic accuracy studies, Quality Assessment of Diagnostic Accuracy Studies - 2 (QUADAS-2), was used. As recommended, we prepared topic-tailored definitions for index questions given along four domains (A-D).

**Domain A. Patient selection**

**1. Risk of Bias**

- A1 Was a consecutive or random sample of patients enrolled?

Yes: consecutive or random enrollment from all sites of recruitment within the recruitment period

No: selected patients

Unclear: no description

- A2 Was a case-control design avoided?

Yes

No

- A3 Did the study avoid inappropriate exclusions?

Yes: justified exclusion: conditions imposing an extra risk of variceal screening endoscopy (e.g., pregnancy), or interfering with elastography (e.g., ascites or extreme obesity) or with the applicability of Baveno criteria (e.g., parenchymal or portal decompensation), previous variceal bleeding, pharmacological or mechanical therapy for portal hypertension, malignant tumors (e.g., hepatocellular carcinoma), splanchnic thrombosis, liver transplantation, non-cirrhotic portal hypertension, splenectomy.

No: unjustified exclusions: viremic status, use of direct-acting antiviral drugs (DAAs), patients with known varices, or rare etiologies (e.g., hemochromatosis or Wilson’s disease).

Unclear: no description

- Algorithm to summarize risk of bias*:

1. 3 x Yes => Low risk of bias
2. at least one No => High risk of bias
3. otherwise => Unclear risk of bias

**2. Applicability**

Low concern: includes patients with suspected or definitive compensated advanced chronic liver diseases (cACLD) (as per defined by The Baveno VI Consensus Workshop)

High concern: any conditions opposing the criteria of cACLD

Unclear concern: no description

**Domain B. Index test - transient elastography (TE) and platelet count measurement**

**1. Risk of bias**

- B1 Were the index test results interpreted without knowledge of the results of the reference standard?^*^

Yes: the operator measuring liver stiffness is blinded to the results of variceal screening endoscopy

No: no blinding

Unclear: no description

- B2 If a threshold was used, was it pre-specified?

Yes: pre-specified liver stiffness > 20 kPa and platelet count < 150 000 /µl

No: no pre-specified cut-off

Unclear: no description

- Algorithm to summarize risk of bias:

1. 2 x Yes => Low risk of bias^*^
2. at least one No => High risk of bias
3. otherwise => Unclear risk of bias

^*^Review authors think that lack of blinding does not impose a substantial risk of bias because of the objective nature of liver stiffness measurement. Therefore, an Unclear + Yes combination was accepted as Low risk of bias.

**2. Applicability**

Low concern: transient elastography was performed by adhering to the current guidelines with detailed description provided about the procedure (the number of valid measures, operator’s experience, prior food deprivation, etc.)

High concern: transient elastography is not performed as per the recommendations

Unclear concern: no description

**Domain C. Reference standard - variceal screening endoscopy**

**1. Risk of bias**

- C1 Is the reference standard likely to correctly classify the target condition?

Yes: variceal screening endoscopy for all patients

No: missed variceal screening endoscopy for any patient

Unclear: no description

- C2 Were the reference standard results interpreted without knowledge of the results of the index test?

Yes: endoscopists were blinded to liver stiffness

No: no blinding

Unclear: no description

- Algorithm to summarize risk of bias:

1. 2 x Yes => Low risk of bias^*^
2. at least one No => High risk of bias
3. otherwise => Unclear risk of bias

^*^Review authors think that lack of blinding does not impose a substantial risk of bias because of the objective nature of varix detection. Therefore, a Yes + Unclear combination is accepted as Low risk of bias.

**2. Applicability**

The domain was divided into two sections:

- Esophageal varices needing treatment

Low concern: graded by visual inspection (size and morphology) with or without insufflation on variceal screening endoscopy on a 2- or 3-grade scale. Varices needing treatment were defined as medium/large esophageal or the presence of red signs on EVs with any grade.

High concern: deviations from that mentioned above (e.g., grading upon insufflation exclusively, handling gastric and esophageal varices together).

Unclear concern: no description

**Domain D. Flow and timing**

**1. Risk of bias**

- D1 Was there an appropriate interval between transient elastography and variceal screening endoscopy?

Yes: ≤12 months

No: >12 months

Unclear: no description

- D2 Was there an appropriate interval between the measurement of platelet count and variceal screening endoscopy?

Yes: ≤12 months

No: >12 months

Unclear: no description

- D3 Did all patients receive a reference standard?

Yes: all patient underwent variceal screening endoscopy

No: some patients did not undergo variceal screening endoscopy

Unclear: no description

Algorithm to summarize risk of bias:

1. 4 x Yes => Low risk of bias
2. at least one No => High risk of bias
3. otherwise => Unclear risk of bias
