# Supplementary material for: Baveno Criteria Safely Identify Patients With Compensated Advanced Chronic Liver Disease Who Can Avoid Variceal Screening Endoscopy: A Diagnostic Test Accuracy Meta-Analysis
Source: Front Physiol. 2019 Aug 13;10:1028. doi: 10.3389/fphys.2019.01028 (PMC6711320; doi:10.3389/fphys.2019.01028)
Supplement: Supplementary Appendix 3 — Excluded studies with rationales of exclusion. [file Table_3.docx]

**Excluded on full-text assessment (or on abstract assessment in the case of conference materials)**

| **Study** | **Reason for exclusion** |
| --- | --- |
| Bota et al. 2015, Bursics et al. 2017, Ding et al. 2015 (full-texts) | Baveno criteria were not used |
| Augustin et al. 2016, Maurice et al. 2017, de Mattos et al. 2017 (full-texts) | letters without relevant data |
| Cales et al. 2017, Shearer et al. 2018, Calvaruso et al. 2019, Pariente et al. 2019, (full-texts) Ahmed et al. 2016, Poggio et al. 2017, Siu et al. 2017, Ratiu et al. 2019, Fennessy et al. 2018 (abstracts) | patients with hepatic decompensation are/may be included |
| Paternostro et al. 2015, Rotaru et al. 2017 (abstracts) | data on varices needing treatment are not reported |
| Vlachogiannakos et al. 2018 (abstract) | shear wave elastography was used |

**Complete overlaps resulting in complete exclusion**

| **Study excluded** | **Study included** |
| --- | --- |
| Fernandez et al. 2015 (abstract) | **Perazzo et al. 2015 (abstract)** |
| Petta et al. 2018 (abstract) | **Petta et al. 2018 (full-text)** |
| Turco et al. 2016 (abstract) | **Jangouk et al. 2017 (full-text)** |
| Tadkalkar et al. 2018a and 2018b, Devadas et al. 2018 (abstracts) | **Tadkalkar et al. 2018c (abstract)** |
| Tosetti et al. 2016, 2017, and 2019 (abstracts) | **Tosetti el al. 2019a and 2019b (full-text and abstract, respectively)** |
| Moctezuma-Velazquez et al. 2017 (abstract), Tasayco et al. 2018 (abstract) | **Moctezuma-Velazquez et al. 2019 (full-text)** |
| Dajti et al. 2019 (full-text) | **Colecchia et al. 2018 (full-text)** |
| Kang et al. 2018 (abstract), Lee et al. 2018a (abstract) | **Lee et al. 2018b (full-text)** |
| Berger et al. 2019 (abstract), Merchante et al. 2018 (abstract) | **multiple overlaps with the included Italian and France cohorts of patients** |

**Partial overlaps resulting in partial exclusion of multicenter studies**

| **Multicenter study (partially included)** | **Overlapping included studies** |
| --- | --- |
| **Augustin et al. 2017** (1 cohort included out of 3 cohorts) | Maurice et al. 2016 |
| **Petta et al. 2018** (7 centers included out of 10) | Colecchia et al. 2018  Tosetti et al. 2018a and 2018b |
