# Supplementary material for: Baveno Criteria Safely Identify Patients With Compensated Advanced Chronic Liver Disease Who Can Avoid Variceal Screening Endoscopy: A Diagnostic Test Accuracy Meta-Analysis
Source: Front Physiol. 2019 Aug 13;10:1028. doi: 10.3389/fphys.2019.01028 (PMC6711320; doi:10.3389/fphys.2019.01028)
Supplement: Supplementary Appendix 4 — Publication bias: funnel plots and results of the Egger's tests. [file Table_4.docx]

Small-study effect across studies including patients with chronic liver diseases.

**A. Missed VNT rate among cCLD patients**

 Egger's test: p=0.183

**B. Spared endoscopy rate among cCLD patients**

 Egger's test: p=0.738

**C. Missed VNT rate among cACLD patients**

 Egger's test: p=0.391

**D. Specificity**

 Egger's test: p=0.319
